# Antifungal Drug Resistance: An Emergent Health Threat

**DOI:** 10.3390/biomedicines11041063

**Published:** 2023-03-31

**Authors:** Antonio Vitiello, Francesco Ferrara, Mariarosaria Boccellino, Annarita Ponzo, Carla Cimmino, Emilio Comberiati, Andrea Zovi, Salvatore Clemente, Michela Sabbatucci

**Affiliations:** 1 Directorate General for Health Prevention, Italian Ministry of Health, Viale Ribotta 5, 00144 Rome, Italy; 2 Pharmaceutical Department, Asl Napoli 3 Sud, 80059 Naples, Italy; 3 Department of Precision Medicine, University of Campania “Luigi Vanvitelli”, 80138 Naples, Italy; 4 Department of Biology and Biotechnology, University of Pavia, 27100 Pavia, Italy; 5 Directorate General for Hygiene, Food Safety and Nutrition, Ministry of Health, Viale Giorgio Ribotta 5, 00144 Rome, Italy; 6 Department Infectious Diseases, Italian National Institute of Health, Viale Regina Elena 299, 00161 Rome, Italy

**Keywords:** antifungal, drugs, fungal infections, mycosis, antimicrobial resistance, molecular mechanisms

## Abstract

Fungal infections, named mycosis, can cause severe invasive and systemic diseases that can even lead to death. In recent years, epidemiological data have recorded an increase in cases of severe fungal infections, caused mainly by a growing number of immunocompromised patients and the emergence of fungal pathogenic forms that are increasingly resistant to antimycotic drug treatments. Consequently, an increase in the incidence of mortality due to fungal infections has also been observed. Among the most drug-resistant fungal forms are those belonging to the *Candida* and *Aspergillus* spp. Some pathogens are widespread globally, while others are endemic in some areas only. In addition, some others may represent a health threat for some specific subpopulations and not for the general public. In contrast to the extensive therapeutic armamentarium available for the antimicrobial chemotherapeutic treatment of bacteria, for fungal infections there are only a few classes of antimycotic drugs on the market, such as polyenes, azoles, echinocandins, and a few molecules are under trial. In this review, we focused on the systemic mycosis, highlighted the antifungal drug compounds available in the pipeline, and analyzed the main molecular mechanisms for the development of antifungal resistance to give a comprehensive overview and increase awareness on this growing health threat.

## 1. Fungal Infections

Infections caused by fungal pathogens (named mycosis) are responsible for superficial or cutaneous mycoses, which in general require simple pharmaceutical treatment, and systemic invasive mycoses, which can be severe [1]. In recent years, epidemiological data have recorded worldwide an increase in cases of severe fungal infections, accounting for over 150 million cases per year and resulting in around 1.7 million deaths annually [2]. Invasive mycoses are a rising problem worldwide due to the increasing incidence of cases, especially in the last decade, and the emergence of forms of fungal pathogenic microorganisms that are increasingly resistant to antifungal treatments. Moreover, the ease of microorganism diffusion due to global trade, high frequency of travel, and climate change make fungal infections hardly controllable. Fungi can survive on surfaces for weeks. Of note, some of the disinfectants commonly used in hospitals often are not effective. For example, ethanol 70% was completely ineffective as an antifungal agent against common airborne fungal genera [3], while it was suitable for cleaning small spills against *Candida auris* [4].

Serious fungal infections mainly occur in immunosuppressed individuals, such as patients infected by the Human Immunodeficiency Virus (HIV) or Severe Combined Immunodeficiency (SCID), individuals with endocrine-metabolic disorders, or those undergoing antineoplastic chemotherapy or immunosuppressive therapy after organ transplantation [3]. Fungal infections pose a risk for critically ill patients in healthcare facilities. Patients hospitalized with severe COVID-19 are at risk of healthcare-associated infections, including candidemia, and various fungal infections in COVID-19 patients have been reported worldwide [5,6]. In particular, aspergillosis, cryptococcosis, and zygomycosis (mucormycosis) are overall the most common systemic mycoses. The main risk factors associated with systemic mycosis infections include critical illness, neutropenia, solid tumor, glucocorticoid therapy, diabetes, and age. The increased risk of severe invasive systemic mycoses in immunocompromised patients highlights the importance of immune defenses against commensal microorganisms [7,8]. Among the fungal pathogens most responsible for severe systemic mycoses are strains of *Candida albicans*, *Candida glabrata*, and *Aspergillus* spp. In particular, the fungus of the *Aspergillus* species is very persistent in the hospital environment causing a wide range of infections including life-threatening systemic ones especially in patients with severe immune system impairment. Clinical data indicate that other fungal families are responsible for serious systemic mycoses, as in the case of *Trichosporon*, reported mainly in patients with hematological diseases [9], but also Zygomycetes, *Fusarium, and Scedosporium* spp. [9,10]. In clinical practice, an accurate, early and timely diagnosis combined with effective antifungal drug treatment are of paramount importance for the proper management of systemic fungal infections to avoid serious consequences in patients. Early diagnosis of invasive fungal infections is the central challenge in routine clinical practice and forms the fundamental basis for targeted treatment [11,12,13]. The diagnosis of an invasive fungal infection is based on three elements: clinical examination, imaging and microbiological confirmation/proof of the causative agent. In the case of clinical suspicion of systemic fungal infection, confirmation is almost exclusively by blood culture. Recently, the World Health Organization (WHO) developed the first fungal priority pathogens list (FPPL) that includes the 19 fungi representing the greatest threat to public health divided into three categories: critical, high and medium priority [14]. The critical priority group includes four pathogens: *Cryptococcus neoformans, C. auris, Candida albicans* and *Aspergillus fumigatus*. The high priority group includes seven pathogens: *Nakaseomyces glabrata* (*Candida glabrata*), *Histoplasma* spp., eumycetoma causative agents, *Mucorales*, *Fusarium* spp., *Candida tropicalis* and *Candida parapsilosis.* The medium priority group includes eight pathogens: *Scedosporium* spp., *Lomentospora prolificans*, *Coccidioides* spp., *Pichia kudriavzeveii* (*Candida krusei*), *Cryptococcus gattii*, *Talaromyces marneffei*, *Pneumocystis jirovecii* and *Paracoccidioides* spp. Some of these fungal pathogens (e.g., *Paracoccidioides* spp.) are confined to certain geographical areas and therefore are not considered a priority globally. However, in the areas where these pathogens are endemic, they are associated with a significant burden of disease. In addition, some pathogens cause infection and represent a health threat in specific populations only; e.g., *Pneumocystis jirovecii* is one of the main pathogens causing opportunistic infections in people living with HIV/AIDS, but it ranked low in the FPPL [14]. All the 19 pathogens lack comprehensive information on the burden of disease, in terms of data from formal surveillance and linkage to clinical outcomes, and susceptibility, mostly from Low–Middle Income Countries (LMIC), likely due to limited access to medical mycology laboratories in resource-limited settings.

## 2. Systemic Mycosis and Pharmacological Therapy

The drugs used in the treatment of fungal infections are traditionally divided into two distinct groups: drugs for systemic use and drugs for topical use. However, this classification is not always applicable since some drugs (imidazole, triazoles, and polyenes) can be used both topically and systemically. Indeed, many superficial mycoses can be treated with both posology. The antifungal chemotherapy of systemic fungal infections has been enriched in recent years by a number of innovative and active, broad-spectrum compounds with a discrete therapeutic index, although greater efforts must be made by the scientific world to increase the therapeutic armamentarium available to clinicians [15]. To date, three main classes of antimycotics are used for treating invasive fungal infections: polyenes, azoles, and echinocandins. Among the polyene antimycotics, amphotericin B is the most potent drug currently available for the treatment of systemic fungal infections, and the reference compound. Amphotericin B has fungicidal and fungistatic activities against numerous fungal pathogens responsible for systemic fungal infections, such as *Candida albicans, Cryptococcus neoformans, Histoplasma capsulatum*, and *Aspergillus*. It is frequently used to treat recurrent fungal infections in patients with compromised defense mechanisms (e.g., patients on immunosuppressive therapy, chemotherapy, and AIDS patients). The drug binds to sterols, particularly to the ergosterol contained in the membrane of susceptible fungi, creating pores from which vital cellular substances escape [16]. Resistance to amphotericin B can occur when structural modifications lead to the reduction in ergosterol in the membrane. Due to the significant toxic effects, patients undergoing antifungal therapy with amphotericin B should be hospitalized at least in the early period of therapy. A preliminary dose administration should provide the degree of reaction of the patient by evaluating the severity of undesirable effects in order to define a therapeutic regimen suitable for the individual and possibly the use of preventive therapy. The effects are generally classified into two groups: infusion-related toxicity and delayed toxicity. To date, amphotericin B remains the drug of first choice in the treatment of systemic fungal infections. The absorption of amphotericin B in the gastrointestinal tract is negligible. Because of the strong bond of the drug to the human tissues, its half-life is approximately 2 weeks. In recent years, two lipid-based formulations developed for oral administration, named amB lipid complex (ABLC) and liposomal amphotericin (L-AmB), have been authorized, characterized by greater selective toxicity towards sensitive fungi, and increased tolerability especially in terms of nephrotoxicity. Indeed, these lipidic formulations interact with ergosterol, leading to increased permeability to univalent and divalent cations and fungal cell death [17]. The most widely used triazoles in clinical practice are itraconazole, posaconazole, and voriconazole; other agents in the class are fluconazole and isavuconazole [18]. Fluconazole inhibits the synthesis of sterol present in the fungal membrane, as it hampers the conversion of lanosterol to ergosterol by inhibition of the enzyme 14-alpha-demethylase, causing increased cell permeability and subsequent loss of cellular constituents. Fluconazole finds clinical indication mainly against *Candida* spp. and *Cryptococcus* spp., both in prophylaxis and therapy. Fluconazole is the drug of choice for treatment of cryptococcal meningoencephalitis pathologies that occur in immunocompromised patients; however, the drug performed poorly compared to other azoles for the treatment of progressive disseminated histoplasmosis (PDH), a serious fungal infection that affects people living with HIV [19]. Fluconazole is excreted renally and presents toxic effects of lower intensity than ketoconazole. The most common side effect pertains to gastrointestinal disorders [20]. Isavuconazole, on the other hand, is indicated mainly in the treatment of invasive aspergillosis and in the treatment of mucormycosis. Isavuconazole is available as intravenous or oral formulations, and it shows less drug–drug interactions and decreased toxicity [21]. Like isavuconazole, posaconazole activity against Mucorales is species dependent, and it can be used as a salvage therapy option for patients who are nonresponsive to other treatments [22,23]. Posaconazole is generally well tolerated, except for minor gastrointestinal side effects due to oral administration. Itraconazole is the most potent triazole compound available. It has a broader spectrum of action than fluconazole, including all *Candida* and *Aspergillus* species, and other rare fungal infections such as *Blastomyces, Cryptococcus,* and *Sporothrix*. Specifically, itraconazole is indicated in the prophylaxis of systemic invasive mycoses caused by aspergillosis in patients with leukemia or bone marrow transplantation. It is also effective against oropharyngeal candidiasis, esophageal and vaginal as well as onychomycosis, griseofulvin-resistant roundworms (worms). Itraconazole is well absorbed after oral administration, is distributed in many tissues including bone and adipose. Itraconazole is metabolized in the liver, and its plasma levels of vary considerably between subjects. Hepatotoxicity can occasionally occur which can be controlled by reducing drug doses [24]. Voriconazole is an antifungal drug used in the treatment or prophylaxis of invasive aspergillosis and candidiasis [25]. Serum concentrations of voriconazole can have considerable interpatient variability, with demonstrated higher hepatotoxicity rate in Asian vs. non-Asian populations [26]. A systematic review and meta-analysis selected the optimal trough concentration of voriconazole for adult patients with invasive fungal infections between 1.0 and 4.0 μg/mL [27]. Finally, echinocandins are a recently marketed class of antifungals, including drugs such as anidulafungin, caspofungin, and micafungin. These antifungals have a unique mechanism of action among antifungal drugs, making them attractive. Echinocandins inhibit the fungal wall synthesis selectively by blocking the activity of β-1-3-D glucan synthetase enzymes, leading to susceptibility of fungal cell to osmotic lysis. Echinocandins are indicated for the treatment of various forms of *Candida* and *Aspergillus* spp. [28]. The WHO has endorsed their importance by adding them to the 2021 Essential Medicines List for both adults and children. 

The sensitivity of the main pathogenic fungi to certain antifungals are detailed in Table 1.

The optimal duration of antifungal therapy is still an unresolved issue depending on several factors, mainly the following: immunological status of the host, type of pathogen and its drug sensitivity, adequateness, and promptness of initial antifungal therapy [29].

## 3. Antifungal Resistance Mechanisms

**Figure 1:**
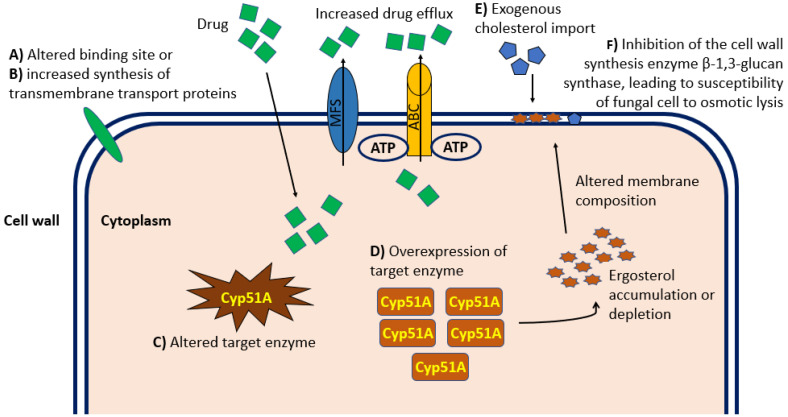
Graphical representation at the cellular level of the principal mechanisms of antifungal drug resistance relevant in the clinical practice. Molecular mechanisms of resistance to antifungal therapy occur mainly through alterations in the cellular binding site (**A**) or increased synthesis of transmembrane transport proteins (**B**), which both lead to reduced intracellular accumulation. In addition, alteration (**C**) or overexpression (**D**) of the enzyme targeted by the drug can cause Ergosterol intracellular accumulation and consequent altered composition and permeability of the cell wall. Import of exogenous cholesterol (**E**) can alter the cell wall composition and permeability resulting in inadequate intracellular drug concentration. Finally, inhibition of the cell wall synthesis enzyme β-1,3-glucan synthase (**F**) can lead to fungal cell susceptibility to osmotic lysis. ABC, ATP-binding cassette transporter family; MFS, major facilitator superfamily of transporters.

Resistance to antifungal drug treatments can be distinguished in intrinsic (primary) forms, which are genetically encoded and associated with fungal species independently of drug exposure, and acquired (secondary) forms, which develop as a consequence of exposure to a certain factor, often an antifungal drug or its structural analogue [30]. Fungal resistance to a specific antifungal compound extends to its entire class. Therefore, the resistance to any class of antifungal drugs can significantly limit the patient’s treatment options. One of the causes of the increase in invasive fungal infections include the emergence of pathogenic forms resistant to common antifungal treatments and the limited access to new pharmacological agents. There are no fungal drug-resistance transposons or plasmids that can pass easily between isolates. However, the growing number of antifungal agents that has been in use for at least twenty years increases the risk of the development of resistant microbes. Indeed, over the last decade, the consumption of systemic antifungal agents has increased globally, with a compound annual growth rate of over 6%, and the High-Income Countries (HIC) have become major consumers of antifungal agents [31]. For example, long-term treatment regimens interfere with and complicate the treatment of underlying diseases, reducing compliance and increasing the risk of drug toxicity and resistance. In addition, the ability of fungi to rapidly mutate by adapting to environmental conditions facilitates the emergence of forms resistant to antifungal treatments, with an inevitable increase in minimum inhibitory concentrations (MIC) to be used during therapy. To date, *Candida* spp. are considered among the pathogenic fungi most implicated in posing an urgent threat to global health [32,33]. Although the molecular characterization of the mechanism of resistance of *Candida* forms to antifungal treatment is not fully understood, some evidence associated mutation in ERG11 and TAC1B as being responsible for fluconazole resistance, and it mainly associated mutations in the FKS gene as being primarily responsible for resistance to echinocandin treatment [34], resulting in upregulation of multidrug efflux transporters and reduced sensitivity to glucan synthase. Some evidence shows that a high percentage of *C. auris* isolates are resistant to fluconazole, approximately 30%–50% are resistant to amphotericin B, and a small percentage are resistant to echinocandins [34,35]. In recent years, more and more forms of *Aspergillus* resistant to antifungal treatments with azoles have been identified, causing high morbidity and mortality as well as an increase in resistance of some *Aspergillus* forms to amphotericin B [36]. Furthermore, the ERG6 gene coding for the sterol-methyltransferase enzyme responsible for altering the molecular target of amphotericin B has been identified. Evidence from several studies demonstrated resistant forms of *Aspergillus* spp. in vitro [37], although clear data and studies fully describing the correlation between amphotericin B MIC values and clinical outcomes in certain patient populations are lacking. The molecular mechanisms of resistance to triazoles occur mainly through increased expression of lanosterol 14-alpha demethylase, alterations in the binding site and increased synthesis of transmembrane transport proteins that lead to drug excretion and decreased intracellular accumulation [37]. Figure 1 shows the main mechanisms of resistance to antifungal treatments. 

Systemic fungal infections, such as bacterial infections, should be included in antimicrobial stewardship programs as an essential component, while health policies should ensure equitable and affordable access to quality antifungal agents, mostly in LMIC.

## 4. New Antifungal Agents

The discovery of new compounds with antifungal activity is a crucial factor to counteract the phenomenon of antifungal-resistant infections. To date, the therapeutic armamentarium of antifungal classes is not as extensive as that of the several antibiotics available against the bacterial infections, and there has been no significant progress in the discovery of new antifungal agents in the recent years. Indeed, the vast majority of new antifungal medications approved for use in the past 10 years have been new versions in the same class of existing agents. Numerous agents in advanced stages of development offer novel dosing regimens and mechanisms of action to combat this health threat [38]. Of note, the fungal cell has few morphological and molecular differences from the human cell, which makes it difficult to identify new fungal specific targets associated with low toxicity for the human cells. An interesting approach taken in recent years used nanoparticles or nanoformulations of commercially available antifungals (Table 2) [39,40]. However, there is an urgent need to discover antifungals with innovative modes of action. A promising strategy comes from inhibitors of the human cell membrane efflux proteins, or from compounds that act simultaneously on several target sites. In addition, a new viable strategy is passive immunization with monoclonal antibodies, so as to strengthen the host antifungal immune response [41,42], particularly in immunocompromised patients who are more prone to systemic fungal infections. Finally, three novel antifungal agents, currently in phase II/III clinical trials, might have an important role for the treatment of invasive candidiasis: phase III results showed the efficacy and safety of rezafungin [43], a novel echinocandin with extended half-life (once-weekly intravenous administration), enhanced tissue penetration/residence time, and limited drug–drug interaction potential; ibrexafungerp (phase III clinical trials) and fosmanogepix are two first-in-class antifungal drugs with acceptable oral bioavailability and broad-spectrum activity against *Candida* spp., including *C. auris* and echinocandin-resistant species (Table 2) [43,44]. These three compounds, together with other new products, i.e., VT-1598 and ATI-2307, were found to be effective against candidemia due to *C. auris* [38].

Research and development (R&D) investments should focus on innovative antifungal agents belonging to a novel chemical class, with no cross-resistance to other antimicrobial classes, binding to new cellular targets, and effective against the priority fungal pathogens graded by WHO. Combination therapies should be optimized in order to improve antifungal spectrum and potency by attacking multiple fungal targets as well as to minimize toxicity and prevent further resistance. 

## 5. Discussion

### 5.1. The Non-Negligible Burden of Invasive Fungal Infections

Infections caused by fungal pathogens can cause severe invasive and systemic diseases that can even lead to death. The mortality rates from invasive fungal infections is generally high, ranging around 10–40% depending on the fungus in an optimal healthcare setting. Individuals most susceptible to serious fungal infections have a compromised immune system, such as people with diabetes, HIV, or undergoing antineoplastic therapy. The burden of this rising health threat is negligible neither in terms of deaths nor in terms of healthcare expenditure. The Leading International Fungal Education (LIFE) portal [45,46] estimated around 350,000 deaths per year due to invasive candidiasis only. However, public awareness of invasive fungal diseases is generally low [47], and improvement of continued educational efforts to increase awareness is needed [48]. A recent study highlighted that the mean direct cost per patient with candidemia and invasive candidiasis ranged from USD 48,487 to USD 157,574, whereas the mean direct cost per hospitalization associated with candidemia and invasive candidiasis was from USD 10,216 to USD 37,715, in developed Western countries [49]. For example, in 2017, invasive fungal infections were associated with approximately USD 7.2 billion in direct costs to the USA Healthcare System alone [50]. Of note, during the COVID-19 pandemic, the reported incidence of invasive fungal infections increased significantly among hospitalized patients in many countries worldwide. 

### 5.2. Drug-Resistant Fungal Infections

In the recent years, the emergence of fungal species resistant to antifungal treatments has raised alarm in the scientific world for the emergence of a new health problem, together with antibiotic and antiviral resistance [51]. In October 2022, the WHO released the first-ever list of health-threatening fungi to guide research, development, and public health action to strengthen the global response to fungal infections and antifungal resistance [14]. The causes of the emergence of fungal forms resistant to antifungal treatments in recent decades may involve complex interactions between environmental conditions, virulence factors, and changes in gene expression. First and foremost, inadequate use by consumers and inappropriate prescribing by clinicians; it is essential to administer antifungals after a microbiological diagnosis, according to the local/national recommendations or guidelines, and technical data sheets. Another cause is the patient’s lack of adherence to the therapeutic antifungal treatment, which can cause suboptimal drug concentrations and increase the likelihood of the development of antifungal-resistant fungal species. Furthermore, the use of fungicides in agriculture has probably favored the emergence of drug-resistant forms [52]. Finally, the limited market entry of new antifungals, an enlarging global immunocompromised population (i.e., resulting from cancer, untreated HIV infection/AIDS, and COVID-19) and the wide range of possible hosts, with virtually unlimited international travels and fungal dispersion and survival in the environment contributed to the emergence of antifungal-resistant fungi. Moreover, fungi can form biofilms, with rapid adaptation and evolution through genomic plasticity and both asexual and sexual reproduction. In particular, plastic-associated fungal communities in the natural environment—the ‘plastisphere’—often differ from the microbial communities in the surrounding area and might favor intraspecies interactions and even increase the natural evolutive pressure toward the development of resistance mechanisms [53]. Usually, the genetic alterations that confer to fungal species a reduced sensitivity to antimycotics are point mutations and genome rearrangements. In addition, nongenetic mechanisms of resistance including tolerance and hetero-resistance are recognized as responsible for antifungal drug treatment failure associated with relapsing fungal infections. Only a few classes of antifungals are available to clinicians, and in many cases, their use is limited by dangerous drug–drug interactions in patients on polypharmacological therapy [54]. If the molecular mechanisms that give certain fungal species resistance to antifungal treatment are well known for all the major drug classes, the complex biological and physiological factors that promote these mechanisms remain to be clarified and understood. In particular, antifungal resistance associated with *Cryptococcus neoformans*, *C. auris*, and *Aspergillus fumigatus* are of great concern. Epidemiological evidence indicates some resistance to azole treatment among *Candida* and *Aspergillus* species. Isolates of *C. auris* showed reduced ability to respond to fluconazole, amphotericin B, voriconazole, and caspofungin. Additionally, other pathogens are highly antifungal resistant, e.g., *Lomentospora prolificans*, *Fusarium* spp., *Mucorales*, and *Scedosporium* spp. [55,56].

### 5.3. Overall Efforts in the Fight of Severe Fungal Infections

Clinical success in eradicating fungal infections requires early diagnosis and availability of timely and effective antifungal drug treatment for the benefit of patient health and savings in the health systems. The Global Action Fund for Fungal Infections (GAFFI) provided maps showing the availability of the main antifungal drugs per country, how many generic varieties exist, as well as their price range [57]. The majority of antifungal treatment guidelines are informed by limited evidence, as fungal infections receive a very low proportion of all infectious disease research funding. R&D investment in this area should be strengthened, mostly addressing the fungal pathogens that are highly antifungal resistant. The overall amount of investments and projects dedicated worldwide to fungal pathogens are significantly lower than those dedicated to bacterial pathogens. In particular, as of February 2023, in the ten-year period 2017–2026, the total amount of investment geared toward preparedness against fungal pathogens (in terms of basic research, therapeutics, diagnostics, vaccine, capacity building and operational activities, and policies) accounted for less than USD 0.5 billion worldwide, with a total of 73 funders and 1165 projects, while over USD 8 billion from 193 funders and 9501 projects was targeted toward bacterial pathogens [58]. The goal of an adequate antimicrobial resistance program is crucial to preserve and enhance the pharmacological efficacy of the agents used and minimize toxicity to improve patient clinical outcome. Such a program requires a multidisciplinary approach: first and foremost, the selection of the most appropriate antifungal agent, dosage, timing, and optimal route of administration; the use of diagnostic tests which can accompany antifungal therapy; and compliance with recommendations and guidelines. Public–private partnerships and multicounty collaborative research platforms could be also pursued. For example, due to epidemiological shift towards more resistant *Candida* spp. in the last decade, the European Confederation of Medical Mycology developed an international Candida Registry (FungiScope™ CandiReg) to facilitate contemporary multinational surveillance [59] of invasive infections due to *Candida* spp. Some fungal pathogens are global, whereas some others are endemic to certain areas with lots of variations in the incidence and prevalence of fungal conditions between countries. For these reasons, the WHO asks countries for progress in surveillance activities on pathogenic fungi that can facilitate timely detection, risk assessment and response, and monitoring of emerging resistance. Therefore, WHO encourages regions and countries to collect data at regional, sub-regional, or country level to inform local public health priorities and targeted unmet R&D needs. Of note, the Global Antimicrobial Resistance and Use Surveillance System (GLASS)-FUNGI focuses on the surveillance of invasive fungal bloodstream infections caused by *Candida* spp. [60]. Indeed, gathering reliable epidemiological data through notification of cases to public health authorities, the collection of microbiological isolates, and the exchange of information through the electronic early warning platforms already available at least in Europe, such as the European surveillance portal for infectious diseases (EpiPulse, Available online: https://www.ecdc.europa.eu/en/publications-data/epipulse-european-surveillance-portal-infectious-diseases (accessed on 23 March 2023)), will enable informed and coordinated risk management actions by public health authorities. In addition, there is a need to raise awareness in the healthcare facilities to adapt their laboratory testing strategies as well as to implement enhanced control measures. Identification of some fungal pathogens, e.g., *Candida auris*, requires specialized laboratory methodology, as traditional identification methods may lead to misidentification. In LMIC, a lack of laboratory capability for routine fungal detection and surveillance can lead to delayed microbiologic identification until spread has already occurred. Numerous outbreaks due to *C. auris* infection in the healthcare setting were reported from countries worldwide [61,62]. These hospital outbreaks have been difficult to control despite enhanced control measures. Affordable point-of-care rapid screening tests might help, particularly in LMIC. Some countries lack a national mycology reference laboratory that can assist clinical laboratories with fungal pathogens identification, antifungal susceptibility testing, and molecular typing, as well as support epidemiological investigations. Most of the countries have no information on invasive fungal infections available at the national level. In conclusion, fungal infections and resistance to antifungal treatments are an emerging area of concern for global public health. Considerable progress has been made in this field to date. However, attention must be increased to this topical and crucial issue [63].

### 5.4. Limits of Our Study

Our study has some limitations. We focused on systemic mycosis due to the severity of these infections, with no mention of diagnostic tools for their identification. Moreover, we focused on fungal pathogens of clinical importance, with no mention of the environmental contamination with antifungal agents as well as the global warming emergence hypothesis [64]. 

## 6. Future Perspectives

Fungal diseases can affect anyone, with around 50% of the global cases of candidemia being reported in Asia [45,46,63]. Annually, the Fungal Disease Awareness Week**—**the next one occurring on 1–7 October 2023—highlights the importance of prompt diagnosis and early antifungal stewardship in the course of a patient’s illness to provide lifesaving treatment. Typically, when a sick person may have a fungal infection and is treated with medicines for another type of disease, they do not get better. Therefore, it is crucial that healthcare providers and patients “think fungus” when symptoms do not get better with treatment. The diagnosis of a fungal infection can be more difficult than that of a bacterial infection, and late diagnosis contributes substantially to the health outcome and the economic burden associated with fungal infections. Furthermore, in the context of the COVID-19 pandemic, as symptoms of some fungal diseases can be like those of COVID-19, including fever, cough, and shortness of breath, laboratory testing is necessary to distinguish between a fungal infection or COVID-19. The correct laboratory diagnosis is essential in order to provide appropriate care, avoiding unnecessary prescription of inadequate drugs, prolonged hospitalization, and useless economic expense. The worldwide strategy to combat invasive fungal infections that cause many annual**—**partly avoidable**—**deaths relies on investments to develop new antifungal drugs with novel mechanism(s) of action as well as diagnostics identifying antifungal resistance, both equally accessible in HIC and LMIC.

## 7. Conclusions

The incidence and geographic spread of fungal diseases are both expanding worldwide due to a number of factors: the rising number of immunocompromised patients, emergence of fungal pathogenic forms that are increasingly resistant to antifungal drug treatments, increase in international travel and trade, global climate warming, insufficient diagnostic and laboratory capacity, and lack of awareness and R&D. In addition, life-saving antifungal agents may be underutilized, especially in LMIC, due to scarce health policy coordination, thus not ensuring equitable access to appropriate antifungal agents [65] and data-informed national guidelines. Improvements are needed in terms of laboratory awareness and capacity, evidence-based recommendations, sustainable investments in R&D and innovation, as well as intersectoral and cross-country collaboration. There is also urgency to apply accurate and timely diagnostic methods and surveillance systems enabling rapid alert and fast implementation of appropriate measures in healthcare facilities, as well as to develop new classes of antifungal drugs with innovative mechanism of action that can avoid therapeutic failure and further loss of life. Where appropriate, diagnostic services should be prioritized to serve populations at greatest risk of fungal diseases (e.g., patients with cancer, HIV/AIDS, Tuberculosis, chronic obstructive pulmonary disease (COPD), and asthma). Improved education of prescribers, pragmatic clinical trials to study opportunistic mycoses caused by resistant fungi, together with initiatives that can facilitate and promote research into therapeutics and diagnostics are also crucial to set preventive measures that can help control this rising phenomenon in a timely manner and prevent inter-hospital transmission, including cross-border spread. The emergence of pathogenic fungal forms increasingly resistant to antimicrobial chemotherapeutic treatments and responsible for severe human infections is a global health issue that requires more focus. 

## Figures and Tables

**Table 1:** Main fungal forms in the clinical practice and sensitivity to antifungal treatments are described in the table. High: High sensitivity; Medium: Intermediate sensitivity; Low: Low sensitivity.

Main Fungal Forms in the Clinical Practice	Sensitivity to Main Antifungal Treatments						
	Polyene	Triazoles					Echinocandin
	Amphotericin B	Itraconazole	Voriconazole	Posaconazole	Isavuconazole	Fluconazole	Anidulafungin Caspofungin Micafungin
*Aspergillus fumigatus*	low	low	high	medium	medium	low	high
*Aspergillus hiratsukae*	high	high	high	high	medium	Low	high
*Aspergillus lentulus*	low	low	low	medium	medium	low	high
*Candida* spp.	medium (not against *C. krusei*, *C. lusitania*)	medium/high	species-dependent	medium	species-dependent	species-dependent	medium/high
*Fusarium* spp.	medium	no activity/low	species-dependent	medium	low	low	low
*Fusarium solani*	medium	low	low	low	low	low	low
*Lamentospora prolificans*	low	low	low/medium	low	low	low	low
*Mucorales*	medium	no activity/low	no activity/low	species-dependent	species-dependent	low	low

**Table 2:** New antifungal agents and principal study outcomes.

Antifungal Compound/Agent	Study Outcome	Reference
Nanoparticles/nanoformulations	High efficacy and selectivity index indicated the superiority of the amphotericin nanoformulations.	[39]
	Surface charge, hydrophobicity, and stabilizing agents of nanoparticles can affect the antifungal activities. Long-term use may lead to accumulation in the host’s organs and cause harm. Biodistribution studies are needed before clinical use could be deemed safe. Synergistic fungicidal effects of antifungal drugs with inorganic nanoparticles could reduce dosage of both these agents, thereby reducing toxicity.	[40]
Monoclonal antibodies	Passive immunization with mAbs against *Aspergillus* spp. improved survival in mouse models of invasive aspergillosis.	[41]
	Effectiveness depends by several variables: type of pathogen, antifungal mechanism of action, biological properties of immunoglobulins, routes of experimental infections, prophylactic rather than therapeutic use, optimal immunoglobulin dosage.	[42]
Rezafungin	Rezafungin was non-inferior to caspofungin for the primary endpoints of day-14 global cure and 30-day all-cause mortality. Phase 3 results show the efficacy and safety of rezafungin and support its ongoing development.	[43]
Ibrexafunge, Fosmanogepix	Ibrexafungerp and fosmanogepix are first-in-class molecules and display extended antifungal spectrum, in particular against echinocandin-resistant *Candida* spp. (including *C. auris*).	[44]

## Data Availability

All data stated in this review are available in the References cited.
